# Permanent Laser Scanner and Synthetic Aperture Radar Data: Correlation Characterisation at a Sandy Beach

**DOI:** 10.3390/s22062311

**Published:** 2022-03-16

**Authors:** Valeria Di Biase, Mieke Kuschnerus, Roderik C. Lindenbergh

**Affiliations:** Department of Geoscience and Remote Sensing, Delft University of Technology, 2628 CN Delft, The Netherlands; m.kuschnerus@tudelft.nl (M.K.); r.c.lindenbergh@tudelft.nl (R.C.L.)

**Keywords:** terrestrial laser scanner, SAR, coastal environment, weather effect, surface roughness

## Abstract

In recent years, our knowledge of coastal environments has been enriched by remotely sensed data. In this research, we co-analyse two sensor systems: Terrestrial Laser Scanning (TLS) and satellite-based Synthetic Aperture Radar (SAR). To successfully extract information from a combination of different sensors systems, it should be understood how these interact with the common environment. TLS provides high-spatiotemporal-resolution information, but it has high economic costs and limited field of view. SAR systems, despite their lower resolution, provide complete, repeated, and frequent coverage. Moreover, Sentinel-1 SAR images are freely available. In the present work, Permanent terrestrial Laser Scanning (PLS) data, collected in Noordwijk (The Netherlands), are compared with simultaneous Sentinel-1 SAR images to investigate their combined use on coastal environments: knowing the relationship between SAR and PLS data, the SAR dataset could be correlated to beach characteristics. Meteorological and surface roughness have also been taken into consideration in the evaluation of the correlation between PLS and SAR data. A generally positive linear correlation factor up to 0.5 exists between PLS and SAR data. This correlation occurs for low- or moderate-wind-speed conditions, whilst no particular correlation has been highlighted for high wind intensity. Furthermore, a dependence of the linear correlation on the wind direction has been detected.

## 1. Introduction

During recent years, Terrestrial Laser Scanning (TLS) has been successfully exploited in many applications thanks to its ability to capture both geometric information and to register backscattered laser intensity of the scanned objects. Among its applications, forestry [1,2,3], river systems [4,5] and geomorphology [6,7] have been investigated. In recent years, our knowledge on coastal environments has been enriched by information provided by TLS systems, which show significant potential for examining coastal processes [8,9,10]. Among the coastal applications, TLS has been used in order to generate Digital Elevation Models (DEM) and to evaluate accurate volumetric changes on beaches, dunes and cliffs, Ref. [11]: thanks to the high density of the point clouds with high accuracy/precision, TLSs are suitable for the detailed DEM mapping of features on hundreds of meters of beach–dune systems [12]. Several studies have also demonstrated the potential for estimating other beach features, such as the surface moisture, using both short and long-range TLS [10,13,14,15,16].

TLS has the advantage over other surveying techniques in that it can provide accurate and dense information in a rapid and non-invasive manner [17]. Moreover, it can scan a beach repeatedly without correction for changes in illumination because it works as an active sensor [13,14,15,18]. On the other hand, TLS has some disadvantages when used in large environments such as coastal areas, such as a limited field of view, high economic cost, heavy material (difficulties for portability), longer measurement time, problems with small misalignments requiring calibration of reference points, and sight shadowing [19].

Space-born remote sensing provides a unique ability to monitor and map coastal areas with complete, repeated, and frequent coverage of the Earth’s surface [20]. In particular, active microwave remote sensing systems, despite their lower resolution, can penetrate through clouds and provide continuous and all-weather monitoring. This allows for more reliable and consistent sand monitoring. Synthetic Aperture Radar (SAR) is the most common active remote sensing system for Earth observation [21]. In recent years, many studies have demonstrated the advantage of using SAR for the estimation of soil surface characteristics, such as surface roughness and soil moisture [22,23]. Different sensor configurations, in terms of wavelength, polarization, and incidence angle, allow for the discrimination of various soil parameters, such as surface roughness, soil dielectric constant, and vegetation cover [24,25].

The combined use of TLS and SAR systems has been poorly investigated, and literature is mostly limited to forest fire [26,27] and vegetation [28] estimation. In this work, we present new results from a Permanent terrestrial Laser Scanner (PLS) [29]-based investigation on beach environments. Both geometric information and backscatter laser intensity have been collected from a permanently installed laser scanning device, a Riegl VZ-2000 [30]. The PLS results are compared with simultaneous Sentinel-1 Synthetic Aperture Radar (SAR) images [31] in order to investigate, for the first time, the synchronous use of PLS and radar in beach environments. The purpose of this comparison is the showing of a possible relation between PLS and SAR data: knowing this relation, SAR data could be correlated to beach characteristics assessabled by PLS.

Several studies have shown that remote sensing data on coastal environments are affected by variables such as wind condition and surface roughness. This has been shown independently for both PLS [10,15,16] and SAR data, where the backscattering behaviour depends on the roughness in relation to the wavelength [32,33] and is affected by wind speed and direction [34]. Therefore, notably, meteorological conditions in terms of wind speed and direction, rain, and surface roughness, will be taken into consideration in the evaluation of the correlation between PLS and SAR data.

In terms of the contribution of surface roughness to the correlation between SAR and TLS systems, the focus of this paper is to evaluate the effect of the roughness at a decimeter-scale resolution on the PLS system and, for the first time, its contribution to the relationship between PLS and SAR data.

In [35], Lane states that roughness, as a component of topography, must be dealt with implicitly at the scale of inquiry; depending on the specific range of scales, there is a diversity in characterising and defining the surface roughness. Higher-order roughness representing elevation variations in the field ([36]) has been considered in this work.

One of the most common parameters applied for surface roughness quantification is the standard deviation in a vertical direction from a single mean value (Root-Mean Squared Height, RMSH).

Variations in height at different scales affect this index, therefore RMSH values are commonly derived on a previously detrended surface in order to remove the effect of larger-scale roughness patterns such as slope or curvature and to separate multi-scale effects [37,38].

TLS has been investigated as a technique for two-dimensional sampling of soil heights able to detect elevation differences at the mm range with relatively small effort, despite the high equipment costs [39,40,41]. Assessment of surface roughness is one of the most challenging applications of TLSs [42].

In the present study, roughness patterns at different scales were analysed by RMSH, evaluated using PLS data, using sliding windows. The purpose of this analysis is to verify whether the RMSH index, describing in this study roughness characteristics in the decimetre scale, has an influence in the correlation between TLS and SAR, and how this influence changes with weather condition.

The paper is structured as follows: in Section 2, the study area, the weather data and the PLS and SAR datasets are presented; in Section 3, the data processing and the roughness evaluation are presented and the methodology used to compare PLS and SAR data is shown; in Section 4, the correlation between SAR and PLS on the study area is presented, followed by the evaluation of influence of weather and roughness. Section 5 and Section 6 show the discussion and conclusion of the present work.

## 2. Materials

In this section, the study area and the used weather dataset are described, followed by the presentation of the TLS and SAR dataset.

### 2.1. Study Area

**Figure 1:**
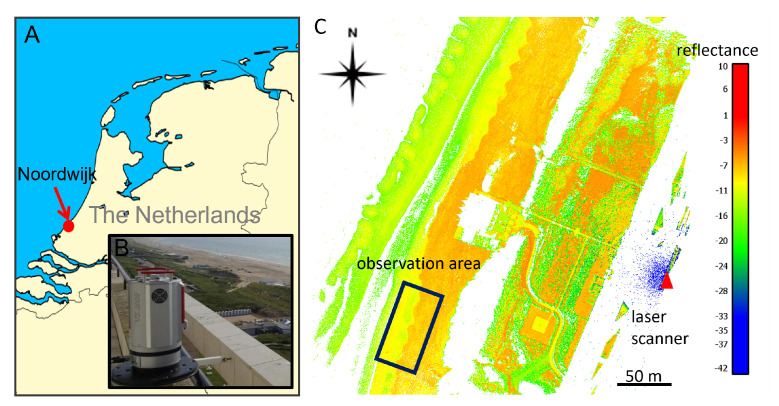
Location of the study site in Noordwijk, The Netherlands (**A**) with view of the Riegl VZ-2000 laser scanner mounted on the balcony of Grand Hotel Huis ter Duin (**B**) and point cloud colored with reflection (**C**) and indication of location of the laser scanner and study site.

**Figure 2:**
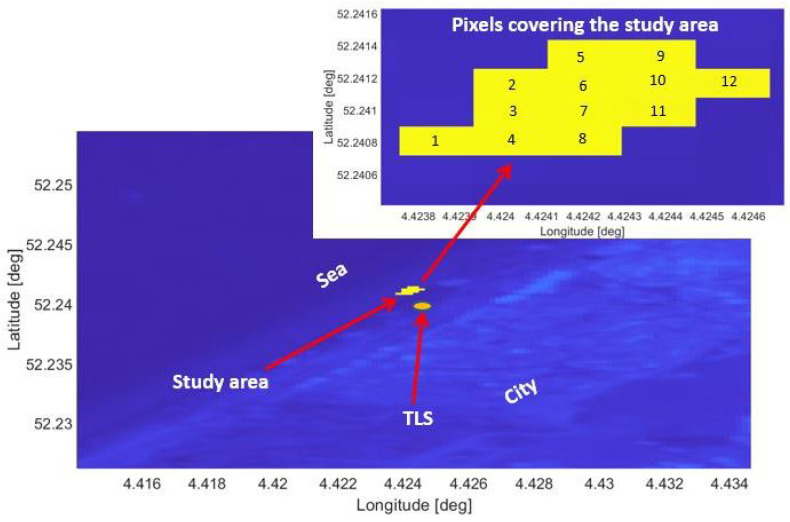
Sentinel-1 image (sigma naught values, VV polarization) from orbit DSC37: the study area is highlighted in yellow and its location is represented with respect to the city, the sea and the PLS position. Top right: the number of the SAR pixels covering the study area.

The study site consists of a typical urban beach on the Dutch coast in Noordwijk, The Netherlands. It is subject to tidal differences up to 2 m and varies in width between 80 m and 140 m under normal weather conditions. Behind the beach lies a row of dunes. A hotel (Grand Hotel Huis ter Duin) borders the dunes at about 150 m distance from the sandy beach. On the balcony of the top floor of the hotel, at 55 m above sea level, a Riegl VZ-2000—shown in Figure 1—laser scanner was installed to acquire point clouds of the sandy beach and neighbouring dunes every hour for the duration of two years. SAR and PLS data on the study area from the same period ranging 19 August 2019 to 22 April 2021 have been identified. Fistly, SAR pixels covering the study area have been selected as shown in Figure 2.

### 2.2. Weather Dataset

Several meteorological stations monitor the Dutch coast, continuously providing detailed variables. Professional weather station data guarantee controlled information but there are no data available close to the study area; therefore in the present study, as a compromise, amateur weather station data have been used. These are less controlled compared to professional weather station data, but are available in the area of interest. For the present study, information regarding wind speed and direction and precipitation at the time of the satellite pass were collected. All the weather data used in this study are local information collected at the moment of the satellite pass. Instantaneous values for both wind speed and direction have been used, whilst for the rain the precipitation accumulation, which is the sum of precipitation over a certain period of time, has been used. In particular, the precipitation accumulation over the 1 h before the satellite pass has been considered. Three amateur weather stations were selected, since they provide the type of data and the temporal sampling required. The meteorological stations are located in Noordwijk (52.25° N, 4.43° E), Katwijk (52.19° N, 4.41° E) and Scheveningen (52.11° N, 4.29° E), all close to beach areas and close to the area of interest (respectively 1 km, 5 km and 15 km). The weather stations in Noordwijk and Scheveningen have high correlation coefficients with each other concerning the wind and rain variables. The wind dataset of station Noordwijk during the two years is not as complete as station Scheveningen, therefore it has been discarded. The dataset of station Katwijk has been discarded since the anemometers—devices used for measuring wind speed and direction—are located next to a building or a wall, therefore their correlation with the two other stations was poor. The values of station Scheveningen have been therefore selected for the present analysis (https://wow.knmi.nl/#919666001s, accessed on 15 November 2021).

**Figure 3:**
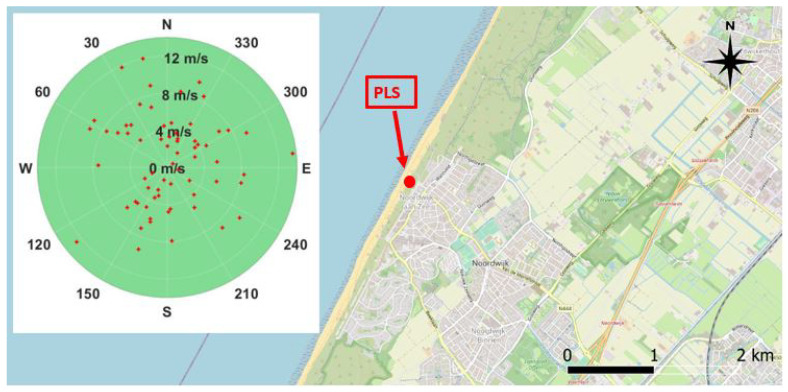
Scatter plot of the local wind direction (0° = North; 90° = East) and wind speed (m/s; represented on the radius) collected during the days of the stack at the moment of the satellite passing. The PLS location is highlighted in red. Background image: QGIS.

Figure 3 shows the scatter of the collected wind speed relative to the wind direction at the moment of the satellite passing for the selected days of the stack. In the figure, each red dot represents the instantaneous wind value (speed and direction) acquired simultaneously to the Sentinel-1 passing over the study area for the entire stack. Considering the orientation of the coastline, onshore wind occurs for directions ranging between −30° and 150°.

### 2.3. Pls Dataset

The selected data consist of point clouds covering an area of approximately 36 m × 80 m on the dry part of the beach, which is not covered by tides under normal conditions. The data are acquired on the same days and at the same time as when the Sentinel-1 passes over the study area. The laser scanner is scanning with 0.03° angular resolution (referred to as low resolution—LR—in the following) and at a wavelength of 1550 nm. The study area is at about a 250 m range distance and the range accuracy (at 150 m range) equals 0.008 m, according to the specifications [43]. With a slight surface slope of 1° towards the sea and away from the laser scanner, the incidence angle is about 77° on average. The area contains just under 30,000 points, resulting in a point density of about 10 points per m^2^. Because of the relatively large incidence angle and range, the footprint in this area is about 0.066 m^2^, with an ellipse shape of 0.3 m diameter on the long side. In the present work, the intensity value is considered for the analysis. The intensity value is normalised with respect to a reference level for each single point, therefore the intensity data are dimensionless [44]. The output has been calibrated to allow the scan data to be range-independent [43].

### 2.4. Sar Dataset

Sentinel-1 is a constellation of two sun-synchronous dawn/dusk orbiting (orbit height: 693 km, platform velocity: about 7.6 km/s) satellites [45], Sentinel-1A and Sentinel-1B, which carry a C-band (operating at a wavelength of about 5 cm wavelength) SAR sensor. The repeat cycle of the Sentinel-1 constellation is 12 days for the single satellites and six days for the two satellites together. Images at different polarisation and resolution are collected and freely accessible from the Copernicus data hub [46].

**Figure 4:**
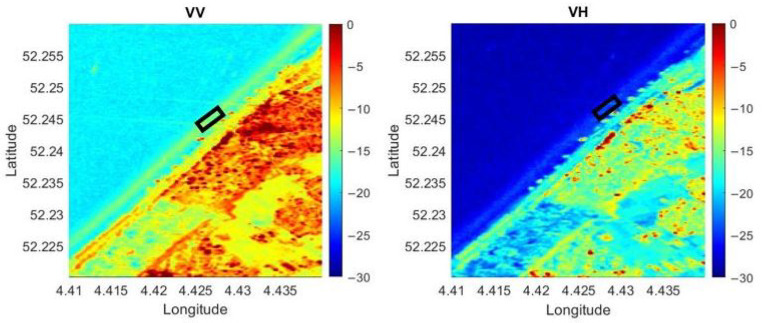
Sentinel-1 images (Left: VV polarisation; Right: VH polarisation): sigma nought values averaged over the period of interest (19 August 2019 to 22 April 2021) from the orbit DSC37 showing the city of Noordwijk and its coast line with the study area highlighted in the black box.

For the present study, Sentinel-1 data collected from Google Earth Engine have been used after further processing steps. A single orbit (DSC37, parallel to the Dutch coastline) has been selected. All the available Sentinel-1 images from the orbit DSC37 acquired from 19 August 2019 to 22 April 2021 at the same time (05:50 UTC) were downloaded (88 images). For Sentinel-1 images, it is possible to select the polarisation of the received signal: VH (vertical transmit, horizontal receive) and VV (vertical transmit, vertical receive) polarisation are available. In Figure 4, the averaged images in both VV and VH polarisation during the period of interest are shown. Regarding the polarisation effect, it is well-known that HH (horizontal transmit, horizontal receive) is more sensitive to surface scattering and VH to volume scattering, and VV a combination of the two. VH backscatter is therefore often used for the retrieval of crop parameters, and HH for ground parameters [47]. It has been shown that HH data are optimal for mapping surface water features when little to no roughness is present across the water surface. Alternatively, HV demonstrates superior results for water surfaces with increased roughness because of high winds [48].

The Sentinel-1 mission only provides data in VV and VH polarisation.

Sentinel-1 imagery in Earth Engine consists of Level-1 Ground Range Detected (GRD). GRD are focused SAR data that have been detected, multi-looked and projected to ground range using an Earth ellipsoid model. The SAR images used in the current study are Interferometric Wide (IW) acquisition mode with 20 × 12 m spatial resolution (range × azimuth), dual polarisation (VV + VH) and GRD product type. Within this collection, all products have been already pre-processed using the European Space Agency’s (ESA) Sentinel-1 Toolbox (S1TBX), available on the European Space Agency Sentinel-1 Toolbox website [49], with the following steps:

- Step 1: Apply orbit file;
- Step 2: GRD border noise removal;
- Step 3: Thermal noise removal;
- Step 4: Radiometric calibration (calculation of sigma nought values);
- Step 5: Terrain correction (ortho-rectification).

Once the stack of images for the area and period of interest are created, three further steps have been performed:

- Step 6: Normalisation of the backscatter coefficients, performed by using a dedicated algorithm. The backscatter of a specific area with a small incidence angle returns higher backscatter values than the data of the same area acquired with a higher incidence angle [50];
- Step 7: Cosine correction. This is the most widely used incidence angle-correction technique [33];
- Step 8: Noise correction. The images of the Sentinel-1 stack have been cropped, including not only the study site, but a bigger area including part of a city, in order to perform noise correction. The pixels with the lowest variability have been evaluated and selected and all the SAR images have been calibrated relative to the low variability area.

## 3. Methods

In the present section, the processing applied to the PLS data in terms of detrending is shown and the methodology to evaluate the roughness index (RMSH) is presented. RMSH is the most common parameter applied in recent studies for quantifying surface roughness [51]. The index is calculated for a regular raster dataset of n × m pixel values.

### 3.1. Pls Data Processing

After data collection with the Riegl VZ-2000 laser scanner, the individual point clouds are transformed into 3D point clouds in compressed laz format in a local coordinate system with the projected location of the laser scanner to elevation zero (NAP, Normaal Amsterdams Peil) as origin. This step is performed in order to obtain positive elevation (NAP) instead of negative values, with respect to the location of the laser scanner at 55 m height. The point clouds are recorded from a fixed location and are therefore already coarsely co-registered (in the order of several centimetres). Fine alignment or geo-location was not deemed necessary for the further analysis for this study. The selected areas of interest are cut out using their x- and y-coordinates and filtered for outliers, i.e., points which are outside of the expected elevation range (mean elevation of the area with a margin of a few decimetres) for the respective areas. Then a plane is fit through the points representing the selected area using principal component analysis (PCA). With the help of the fitted plane, the slope is calculated and removed from the elevation values of the respective areas in order to enable the determination of surface roughness, [37].

### 3.2. Rmsh Evaluation

Surface roughness is reflected by the spatial heterogeneity of elevation values at a pre-defined scale and its quantification depends on the dimensionality and resolution of the data, as well as on the desired expressiveness of the index [51]. Considering that the PLS provides about 10 cm point spacing at the study area, the roughness scale considered in the present work is of the order of magnitude of few decimetres. This scale is the same order of magnitude of the wavelength of the Sentinel-1 C band images used. RMSH is evaluated from a previously detrended surface [37] in order to separate multi-scale effects, with the remaining random roughness representing spatial variations [42,51].

Considering that beach topography is highly heterogeneous in space, a local adaptation of the RMSH, the locRMSH (local RMSH) index, has been applied in the present work. A sliding window calculates the local variations in random roughness. The choice of an appropriate window size is crucial for capturing different surface patterns [51]. The local RMSH is obtained by the following equation [52]:

(1) $$loc\mathrm{RMSH}=\sqrt{\frac{1}{N}\sum_{n=1}^{N}|z_{n}{|}^{2}}$$

with:

- locRMSH, local root-mean squared height;
- *N* the number of points in each cell;
- $z_{n}$, the z-value at the *n*-th points in the window;

In the analysis of the present work, the local adaptation of the RMSH will be used. It will be referred to as RMSH.

#### Choice of Window Size: Comparison between High-Resolution and Low-Resolution Dataset

The selected area was scanned with the above specifications continuously for two years. To investigate the effect of the relatively low resolution (LR) of this dataset on the RMSH evaluation, the same area was scanned with higher resolution (HR) and these data have been compared to the LR data acquired one hour later in order to define the window cell size for the locRMSH estimation. The same laser scanner with 0.015° angular spacing was used on two occasions to acquire a scan of the same area, resulting in a point density of about 43 points/m^2^ and the same footprint size (see Table 1). This leads to more overlapping footprints.

**Figure 5:**
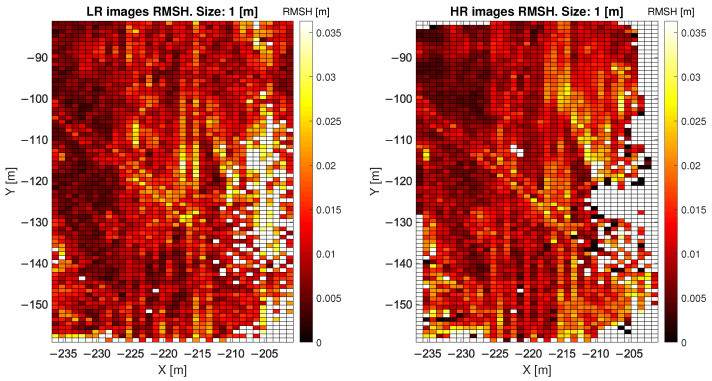
RMSH values evaluated for each pixel with 1 m window size; comparison between HR and LR. The diagonal feature in the middle is a result of tire tracks from bulldozers crossing the study site.

**Figure 6:**
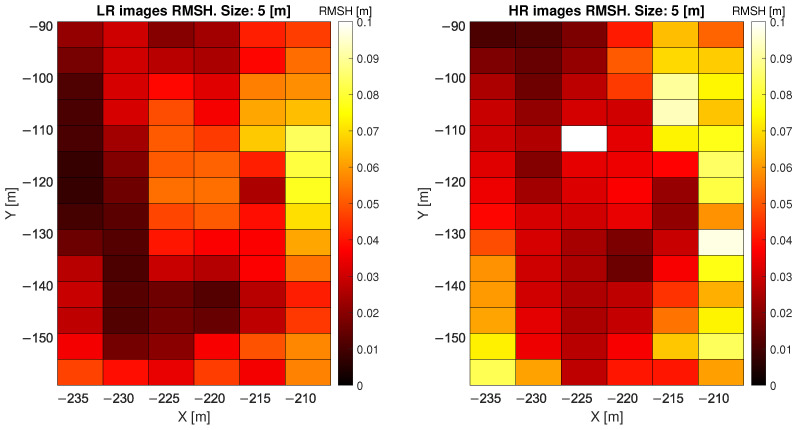
RMSH values evaluated for each pixel with 5 m window size; comparison between HR and LR.

**Figure 7:**
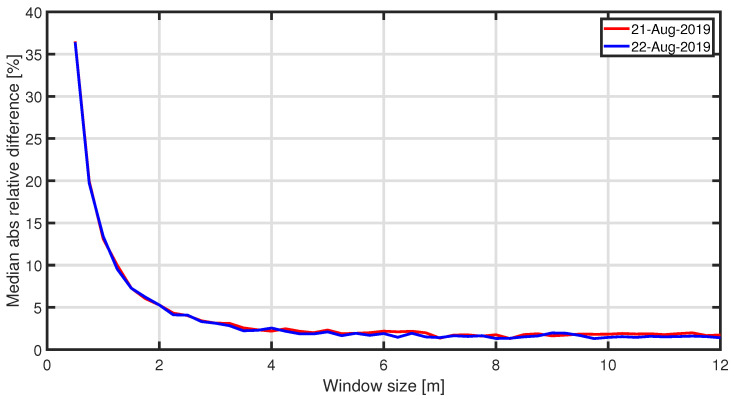
RMSH median relative difference between HR and LR images for the two analysed days when considering different window size, for window size moving from 0.5 m to 12 m.

When considering LR point clouds, the disadvantage of using a window size smaller than 1 m is the low number of points per pixel (less than 11) and that the mean ratio between HR and LR is high, especially when considering a 0.5 m cell. Figure 5 and Figure 6 show, for one of the two considered days of analysis, the value of the local RMSH [m] evaluated in each window cell, measuring 1 m and 5 m, respectively, to give an indication of the order of magnitude of RMSH on the sandy area considered. Figure 7 shows the RMSH median relative difference between HR and LR images when considering different window sizes, for window size moving from 0.5 m to 12 m. This parameter has been computed as follows: for each pixel the relative difference between the locRMSH computed by using the HR image ($loc{\mathrm{RMSH}}_{\mathrm{HR}}$) and the one computed with LR image ($loc{\mathrm{RMSH}}_{\mathrm{LR}}$), indicated by the symbol $\varepsilon_{\mathrm{RMSH}}$, has been evaluated with the equation:

(2) $$\varepsilon_{\mathrm{RMSH}}=\frac{|loc{\mathrm{RMSH}}_{\mathrm{HR}}-loc{\mathrm{RMSH}}_{\mathrm{LR}}|}{loc{\mathrm{RMSH}}_{\mathrm{HR}}}.$$

Then, the median value of $\varepsilon_{\mathrm{RMSH}}$ has been computed. So, Figure 7 shows the median value of $\varepsilon_{\mathrm{RMSH}}$ as a function of the window size for two point clouds, acquired in August 2019. Whilst a significant difference (35%) exists for small window size ( 0.5 m), this difference is almost halved for 1 m cells and converges for window cells ≥4 m, where the difference is about 2%. Therefore, the dimension of the SAR pixels ( 12 m × 20 m) has been used as an RMSH window cell size in the present study, with reduced influence of the laser scanner resolution.

### 3.3. Pls and Sar Comparison

A total of 12 SAR pixels covers the study area, as shown in Figure 2. All the presented analyses have been conducted for these 12 pixels, which have homogeneous coverage (dry sand). Since SAR images are geo-coded, for each pixel the coordinates of the perimeter and of the pixel centre are known. For each PLS scan, all the PLS points included within each SAR pixels’ perimeter have been selected. Their median value has been considered as the PLS intensity of each pixel.

A correlation factor—in the sense of Pearson’s linear correlation coefficient—has been evaluated for each pixel of the study area between SAR backscatter and PLS intensity, averaged over time. Pearson correlation coefficient is a measure of linear correlation between two sets of data (SAR backscatter and PLS intensity in our case) and is the ratio between the covariance of the two variables and the product of their standard deviations.

Similar correlation factors have been retrieved when considering SAR VV and VH polarization in all the performed analyses. Therefore, it has been decided to show in the rest of the present study only the results obtained with VV polarisation.

In order to further investigate the correlation between SAR backscatter and PLS intensity, other variables which might affect the signal (both PLS and SAR) have been taken into consideration. For this purpose, we consider wind speed and direction at the moment of the satellite passing and of the PLS scan of the beach. The wind speed has been considered separately for a first analysis: all the wind speed values have been divided into three categories: low wind (<4 m/s), medium wind (4.1–8 m/s), and high wind (>8 m/s). The correlation factor between PLS intensity and SAR backscatter dataset has been evaluated for each pixel in the three cases of low, medium and high wind speed.

## 4. Results

In this section, we present the results of the present work in terms of correlation between SAR backscatter and PLS intensity, see Section 4.1. The influence of weather phenomena in terms of wind conditions and of the surface roughness on the PLS intensity and on the correlation between PLS intensity and SAR backscatter is further analysed in Section 4.2 and Section 4.3, respectively.

### 4.1. Pls and Sar Correlation

**Figure 8:**
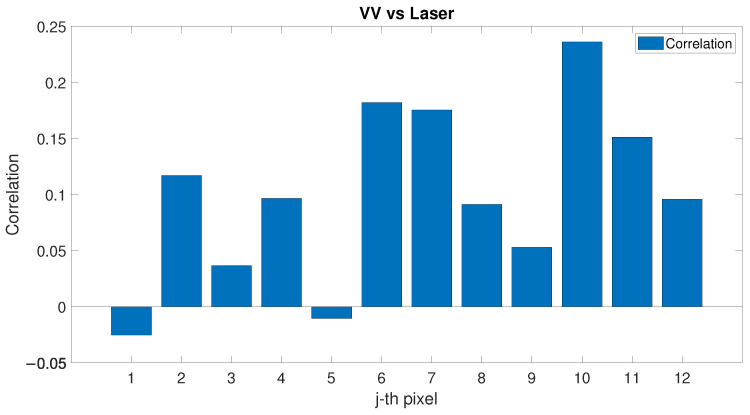
Correlation factor between SAR (VV polarisation) backscatter and PLS intensity evaluated for each pixel of the study area.

**Figure 9:**
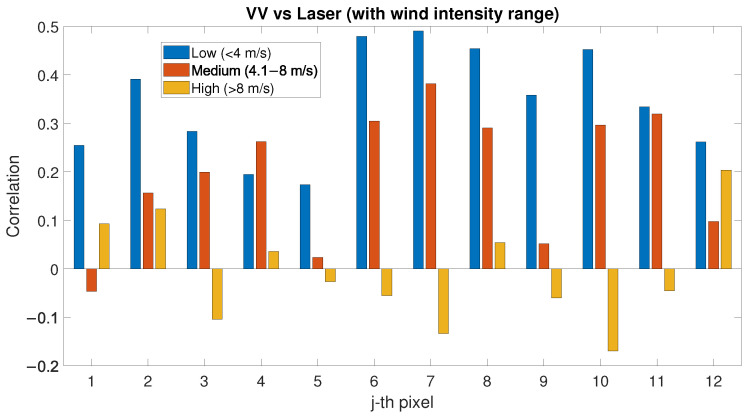
Correlation factor between PLS intensity and SAR (VV polarisation) backscatter evaluated for low-, medium- and high-wind-speed conditions.

**Figure 10:**
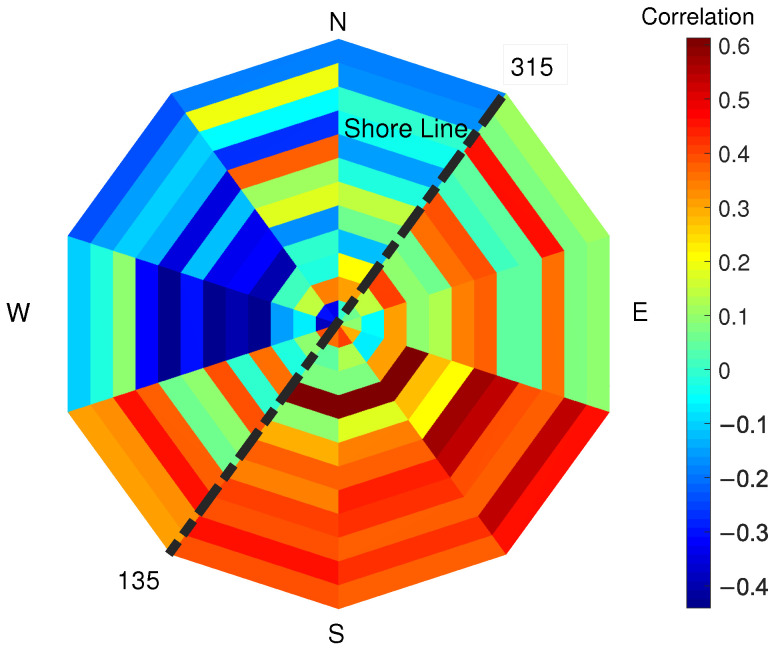
Correlation factor (colour bar) between PLS intensity and SAR backscatter evaluated for different overlapping sectors ranging in 90° wind directions. In the figure, each sector represents a 90° wind-direction section and each of the 12 rings represent a row of pixels starting from pixel 1, which is located on the sea-side (inner ring), and moving towards pixel 12 (external ring), which is located on the city-side of the study area. The dotted black line represents the orientation of the shore line in Noordwijk.

The correlation factor between PLS intensity and SAR backscatter (VV polarisation) evaluated for each pixel of the study area is shown in Figure 8: a generally positive but low correlation between the two variables in each pixel exists. Wind speed and direction at the moment of the satellite passing and of the PLS scan of the beach have been considered to further investigate the correlation between SAR and PLS signals. The correlation factor between PLS intensity and SAR backscatter (VV polarisation) data has been evaluated for each pixel in the three cases of low, medium and high wind speed (see Figure 9). Compared to the previous analysis, the correlation between SAR and PLS data in the separate categories is now always higher and positive for each pixel when considering low or medium wind (up to 0.5 correlation factor). For high wind speed, the correlation becomes lower and irregular, and both positive and negative depending on the considered pixel. To further define the correlation values, the wind directions have also been considered. The correlation factor has been evaluated for different sectors corresponding to different wind directions: each sector ranges by 90° (see Figure 10). In Figure 10, each sector represents a 90° wind-direction section and each of the 12 rings represent a row of pixels starting from pixel 1, which is located on the sea-side (inner ring), and moving towards pixel 12 (external ring), which is located on the city-side of the study area. It is noticed that the correlation has an interesting dependence on the wind direction and different directions show different correlations. In particular, when the wind direction ranges between 90–270°, several pixels show a positive correlation of up to 0.6. For offshore wind (direction ranging between 210° and 360°), most of the pixels have negative correlation up to 0.4. Most of the sectors have similar range of colours, meaning that the 12 pixels of the study area present similar correlations in equal wind direction conditions. The obtained results are shown in Table 2.

### 4.2. Weather Data Effect

A separate analysis has been conducted on the PLS data in order to evaluate to what extent the weather phenomena affect the intensity. In the present study, the same stack of data used in the previous analysis collected over 88 days (acquired between 19 August 2019 and 22 April 2021) has been analysed. During this period, only six days presented an accumulation precipitation bigger than 0 mm/h. These days were considered not sufficient for the statistical analysis. Therefore, only analyses on the influence of wind have been performed.

**Figure 11:**
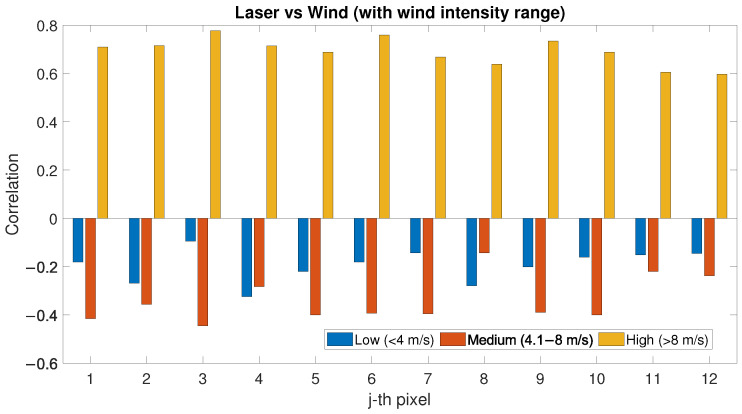
Correlation factor between PLS intensity and wind speed evaluated for low-, medium- and high-wind-speed conditions.

**Figure 12:**
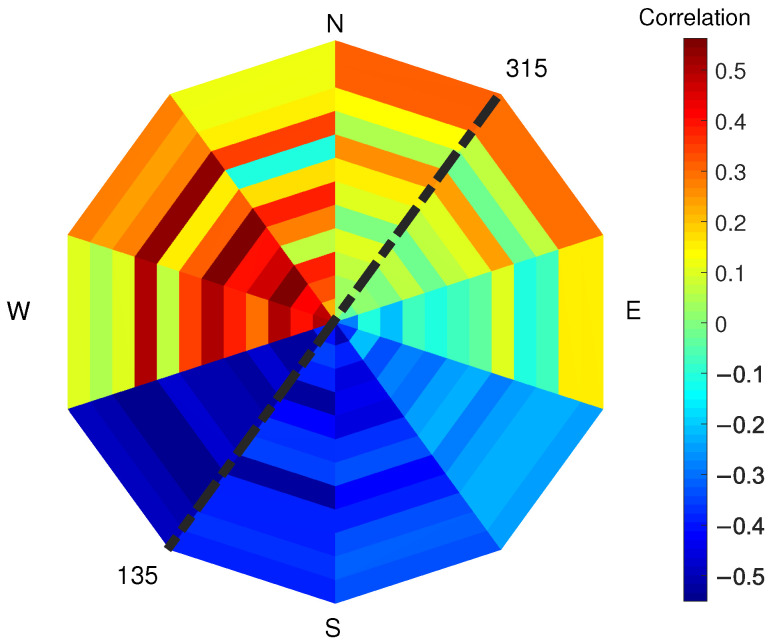
Correlation factor (colour bar) between PLS intensity and wind direction evaluated for different overlapping sectors ranging in 90° wind directions. In the figure, each sector represents a 90° wind-direction section and each of the 12 rings represent a row of pixels starting from pixel 1, which is located on the sea-side (inner ring), and moving towards pixel 12 (external ring), which is located on the city-side of the study area. The dotted black line represents the orientation of the shore line in Noordwijk.

First, wind speed has been considered. Figure 11 shows the correlation factor between PLS intensity and wind speed for low-, medium- and high-wind-speed condition, compared to Section 3.3. For low and medium wind, each pixel shows that the PLS intensity decreases with increasing wind. For high wind speeds, the correlation turns positive and ranges for each pixel between 0.6 and 0.8. This change of sign in the correlation factor between PLS signal and high-wind-speed condition could explain the lack of correlation between PLS and SAR data, as shown in Figure 9. The wind direction has also been considered and the correlation factor between PLS intensity and SAR backscatter has been evaluated for each pixel and for each wind-direction section (See Figure 12). As in the previous section, the correlation factor has been evaluated for different overlapping sectors ranging in 90° wind directions. In the figure, each sector represents a 90° wind-direction section and each of the 12 rings represent a row of pixels starting from pixel 1, which is located on the sea-side (inner ring), and moving towards pixel 12 (external ring), which is located on the city-side of the study area. Again, certain directions show different correlation: in particular, the correlation factor between PLS and wind speed is negative for almost all pixels (up to 0.5) for wind directions ranging between 90° and 300° (mostly onshore wind), whilst a positive correlation exists (up to 0.5) for mostly offshore wind (directions ranging between 210° and 360°). The obtained results are shown in Table 2.

### 4.3. Roughness Influence

**Figure 13:**
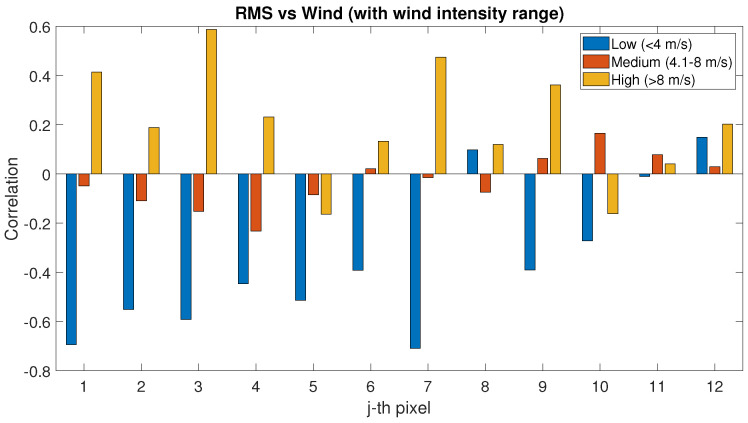
Correlation factor between RMSH and wind speed evaluated for low-, medium- and high-wind-speed conditions.

The roughness variable and to what extent it affects both PLS intensity and SAR signal have been analysed by comparing RMSH index with the TLS and SAR dataset. As mentioned in Section 3.2, the RMSH index has been used as an indication of the roughness of the soil. The index has been locally evaluated for each pixel. As a first step, the correlation between the roughness and the wind has been evaluated (See Figure 13). As explained in Section 3.3, the correlation factor has been evaluated separately for low, medium and high wind speed. For the low-wind-speed condition, the correlation with RMSH is negative for almost all the pixels (up to 0.8); with increasing wind, the RMSH diminishes (lower roughness). For high wind speed, most of the pixels show positive correlation with RMSH (higher roughness).

**Figure 14:**
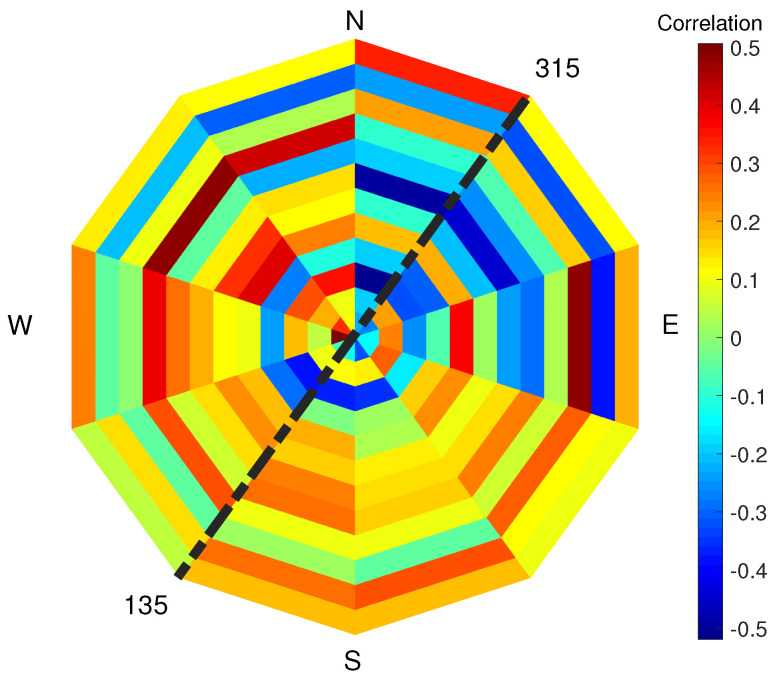
Correlation factor (colour bar) between RMSH and wind speed evaluated for different overlapping sectors ranging in 90° wind directions. In the figure, each sector represents a 90° wind-direction section and each of the 12 rings represent a row of pixels starting from pixel 1, which is located on the sea-side (inner ring), and moving towards pixel 12 (external ring), which is located on the city-side of the study area. The dotted black line represents the orientation of the shore line in Noordwijk.

When considering the wind direction, see Figure 14, no significant correlation seems to exist between wind speed and RMSH, except for a slightly more regular positive correlation in the sectors ranging 90–270°, where there also seems to be a more homogeneous behaviour for the 12 pixels of the area when considering specific wind direction. For other directions, the correlation value is very variable for each considered pixel.

**Figure 15:**
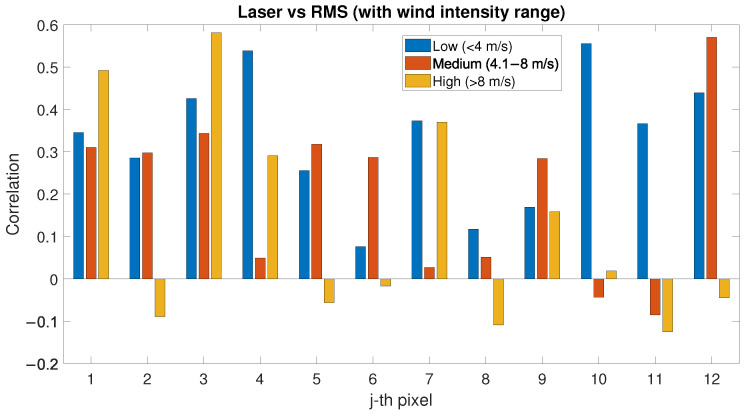
Correlation factor between PLS intensity and RMSH evaluated for low-, medium- and high-wind-speed conditions.

The RMSH values have been compared with PLS intensity and their correlation has been evaluated for low, medium and high wind speed (See Figure 15). Excluding a few cases of high wind conditions for certain pixels, in particular pixels located on the city-side of the study area, an interesting positive correlation exists between PLS intensity and RMSH for each wind speed in all the pixels. In particular, for low wind condition, the correlation is always positive; the cases of negative correlation are limited to a few pixels and to medium–high wind conditions.

**Figure 16:**
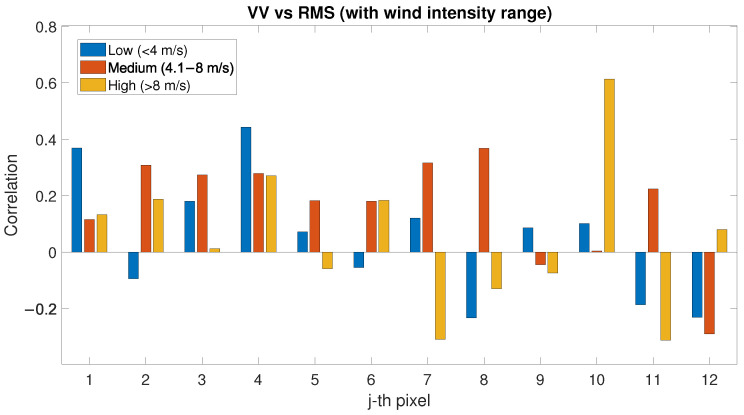
Correlation factor between SAR (VV polarisation) and RMSH evaluated for low-, medium- and high-wind-speed conditions.

The following analysis shows the comparison between SAR signal (VV polarisation) and RMSH index for each pixel (See Figure 16). In this analysis, no particular trend can be highlighted in the correlation between SAR and RMSH, which is in general low and very variable considering different pixels. The obtained results are shown in Table 2.

## 5. Discussion

As presented in Section 4, when environmental variables are not considered, SAR and PLS data seem to have a low correlation (see Figure 8). When considering wind speed in the evaluation of SAR backscatter and PLS intensity correlation, a positive correlation is noticed only for low- and medium-wind-speed conditions; for stronger wind, no correlation can be noticed (see Figure 9). The PLS has a negative correlation with low and medium wind speed; for high wind speed, the correlation is high and always positive for each pixel (see Figure 11). This phenomenon could be explained with the following hypothesis:

(1) High wind speed could dry the sand and, as a consequence, the PLS intensity is higher (since low sand moisture values correspond to higher PLS intensity [10,15]);
(2) There is a relationship between the activation of aeolian transport—above a certain wind speed—and the sand particles moving on the beach surface, which could affect the PLS intensity. In fact, the activation of aeolian transport requires wind speeds above certain values [53].

The low correlation between SAR backscatter and PLS intensity for high wind speed can be ascribed to the correlation trend between PLS intensity and wind: when PLS has a negative correlation with wind speed (low and medium conditions), the behaviour of its intensity is similar to the SAR backscatter; for high wind speed, PLS intensity behaviour is reversed and it can no longer be compared to the SAR backscatter (compare Figure 9 and Figure 11).

RMSH seems to have negative correlation with wind speed only for low wind conditions: in this case, the effect of the wind is a reduction in the RMSH. For higher wind values, in particular for 4–8 m/s, no correlation can be noticed, see Figure 13. The hypothesis is that this phenomenon happens because wind speed in that range could produce a smoother profile on the sand surface.

Even if each pixel has a different correlation value, a positive correlation exists between RMSH and PLS intensity, in particular for low/medium wind speed (see Figure 15), whilst the correlation between SAR backscatter and RMSH is low but generally positive (see Figure 16). The hypothesis in this case is that this can be related to the order of magnitude of the RMSH values evaluated in the present work, which might not significantly affect the SAR wavelength. For future studies, direction/orientation of the roughness could be considered for determining correlations with SAR data, as well as SAR systems with higher resolution with respect to Sentinel-1, which can be used and correlated with RMSH evaluated on different window sizes. Roughness indices can be also evaluated on a lower order of magnitude to identify more specific correlations.

The correlation between PLS intensity and SAR backscatter shows specific wind directions where the correlation is particularly relevant. The same occurs for the correlation between PLS intensity and wind speed. The correlation between PLS intensity and wind speed, for low and medium wind speed, is negative; correlation between SAR backscatter and PLS intensity exists only when the correlation between PLS intensity and wind speed is negative. The wind direction where the correlation between PLS and wind speed is minimum (south) is the same than the direction where the correlation between SAR and PLS is maximum. South is also the direction where low and medium winds generally come from (compare Figure 3, Figure 10 and Figure 12).

## 6. Conclusions

In an investigation into the correlation between permanently installed TLS and SAR systems has been conducted on the beach of Noordwijk, TLS data have been compared with simultaneously acquired Sentinel-1 SAR images. The correlation between TLS and SAR systems on sandy environments and the effect of environmental variables on their correlation have been analysed for the first time. This study showed that the correlation between the two considered systems when not considering external variables is positive but low (up to 0.25). When considering wind speed, a higher correlation between TLS and SAR (up to 0.5) exists in the case of low and medium wind speed, whilst no particular correlation could be highlighted for high-wind-speed conditions. In the present study, only linear correlation between the analysed variables has been evaluated. Further analysis can highlight whether different correlations exist (e.g., for higher wind-speed conditions).

The wind direction has also been considered: for directions ranging 90–270°, the entire area is homogeneous and there is a positive correlation between TLS and SAR up to 0.6, whilst for directions ranging 210–360°, the correlation is negative up to 0.4. The correlation between TLS and wind has been separately considered with the following results: for low and medium wind, PLS and wind speed have a negative correlation, whilst for high wind speed, the correlation turns positive and ranges between 0.6 and 0.8. The correlation between TLS and wind speed also depends on the wind direction: for directions ranging 90–300° a negative correlation is shown and for directions 210–360° there is a positive correlation.

The influence of the surface roughness—evaluated in terms of RMSH—has also been considered in terms of correlation between RMSH and wind. For low-wind-speed conditions, the correlation between RMSH and wind is negative up to 0.8; the correlation becomes positive (higher roughness) with the increase in the wind speed. No interesting correlation has been highlighted when considering the wind directions. The analysis of the correlation between PLS and RMSH showed a positive correlation for each wind speed. In the analysis of the correlation between SAR and RMSH instead, no particular trend has been highlighted. In the roughness analysis, one of the main limits is that the RMSH index has been evaluated using PLS data, with decimetre-scale resolution, which might not significantly affect the SAR wavelength. This aspect can be further investigated in future studies.

In conclusion, this preliminary study allowed for the individuation of a first range of conditions where TLS and SAR data present a good correlation. A better knowledge of the scenarios where the correlation between TLS and SAR is applicable, and of the extent of the existing correlation, could allow for the exploitation of the combined use of TLS and SAR advantages, moving from the small scale (TLS) to a world-wide scale (SAR).

## Figures and Tables

**Table 1:** Properties of the dataset from permanent laser scanning used in this study. LR and HR data only differ in angular resolution, point density and number of files. Incidence angle, point density and footprint size are averaged over the study area. In the present study, HR data (two files) collected during two different days have been only used for a comparison with LR data in order to investigate the effect of the resolution on the RMSH evaluation.

Dataset	LR	HR
Wavelength [nm]	1550	1550
Range accuracy at 150 m [m]	0.008	0.008
Angular resolution [°]	0.03	0.015
Incidence angle [°]	77	77
Point density [pt/$m^{2}$]	10	42
Footprint size [$m^{2}$]	0.066	0.066

**Table 2:** Linear correlation coefficients (maximum, minimum and median values within the considered 12 pixels) between the indicated variables for the three considered wind-speed conditions: low (<4 m/s), medium ( 4.1 m/s–8 m/s) and high (> 8 m/s) wind speed.

Variables/Correlation	Low Wind			Medium Wind			High Wind		
	Min	Median	Max	Min	Median	Max	Min	Median	Max
Laser vs. SAR(VV)	0.17	0.35	0.49	−0.05	0.23	0.38	−0.17	−0.04	0.20
Laser vs. Wind	−0.32	−0.18	−0.09	−0.45	−0.39	−0.14	0.60	0.70	0.78
RMS vs. Wind	−0.71	−0.42	0.15	−0.23	−0.03	0.17	−0.16	0.20	0.59
Laser vs. RMS	0.08	0.36	0.56	−0.09	0.29	0.57	−0.13	0.00	0.58
SAR(VV) vs. RMS	−0.23	0.08	0.44	−0.29	0.20	0.37	−0.31	0.05	0.61

## Data Availability

Not applicable.

## Abbreviations

The following abbreviations are used in this manuscript:

TLS	Terrestrial Laser Scanning
PLS	Permanent Terrestrial Laser Scanning
SAR	Synthetic Aperture Radar
DEM	Digital Elevation Models
RMSH	Root-Mean Squared Height
*loc*RMSH	local Root-Mean Squared Height
LR	Low Resolution
HR	High Resolution
HH	Horizontal-transmit and Horizontal-receive
VV	Vertical-transmit and Vertical-receive
HV	Horizontal-transmit and Vertical-receive
VH	Vertical-transmit and Horizontal-receive
GRD	Ground Range Detected
IW	Interferometric Wide
ESA	European Space Agency
S1TBX	Sentinel-1 Toolbox
NAP	Normaal Amsterdams Peil
PCA	Principal Component Analysis
